# Time to Improvement After Corticosteroid Injection for Trigger Finger

**DOI:** 10.7759/cureus.16856

**Published:** 2021-08-03

**Authors:** Daniel Seigerman, Richard M McEntee, Jonas Matzon, Kevin Lutsky, Daniel Fletcher, Michael Rivlin, Mason Vialonga, Pedro Beredjiklian

**Affiliations:** 1 Orthopedic Surgery, Rothman Orthopedic Institute, New York City, USA; 2 Orthopedic Surgery, University of Kansas, Kansas City, USA; 3 Division of Hand Surgery, Rothman Orthopedic Institute, Philadelphia, USA; 4 Orthopedics, Rutgers Robert Wood Johnson University, New Brunswick, USA

**Keywords:** trigger finger, corticosteroid injection, trigger, stenosing tenosynovitis

## Abstract

Purpose

Trigger finger is a commonly occurring hand condition that presents with symptoms of pain, clicking, locking, and catching of the finger. A common non-operative management option is corticosteroid injection. The purpose of this study was to evaluate the short-term patient response to corticosteroid injections for trigger finger.

Methods

The patients of six fellowship-trained orthopedic hand surgeons who underwent a corticosteroid injection for trigger finger between June 2019 and October 2019 were invited to participate in this study. Patients were contacted by phone at one week, two weeks, and three weeks after the injection to complete a questionnaire regarding their pain and triggering symptoms. Medical records were also reviewed to collect basic demographic data.

Results

A total of 452 patients were included in the study. At the final follow-up, 82.4% of patients reported complete pain relief, 16.3% had partial relief, and 1.2% had no relief from their pain. For their triggering symptoms, 65.9% reported complete triggering relief, 30.4% had partial relief, and 3.5% had no triggering relief. It took an average of 6.6 days following injection for patients to experience complete pain relief, and an average of 8.1 days for patients to experience complete triggering relief.

Conclusions

This analysis found that most patients experience relief of pain and triggering at three weeks following corticosteroid injection. The majority of patients experienced some pain relief within the first week following corticosteroid injection, while improvement in triggering appeared to lag behind pain relief.

## Introduction

Trigger finger or stenosing tenosynovitis is a common hand condition with a prevalence of 2-3% in the general population [1-4]. Typically, patients present with symptoms of pain, clicking, catching, and/or loss of active motion of the affected finger [1]. Initial non-surgical treatment options include observation, non-steroidal anti-inflammatory drugs, splinting, and corticosteroid injections [5-9]. For most hand surgeons, the mainstay of non-operative management is a corticosteroid injection [10-14].

Bunnell described corticosteroid injection for trigger finger as early as 1953 [15]. While the exact mechanism by which corticosteroids resolve trigger finger is unclear, they have been shown to be an effective first-line treatment with reported long-term resolution of symptoms in 32-90% of patients [5,11,13,16,17-24]. However, there is limited data on the short-term response to corticosteroid injections for trigger finger [11]. Specifically, minimal information exists regarding the timing of symptom resolution. This would be valuable for counseling patients and for setting appropriate expectations during the office visit when the corticosteroid is administered.

The purpose of the present study was to evaluate the short-term patient response to corticosteroid injections for trigger finger. We hypothesized that single corticosteroid injection is an effective first-line treatment that would provide pain relief within one week and mechanical relief within three weeks. 

## Materials and methods

After obtaining institutional review board approval (Thomas Jefferson University Office of Human Research Institutional Review Board issued approval 13D.432), we identified the patients of six fellowship-trained orthopedic hand surgeons who were treated only with a corticosteroid injection for nodular trigger finger. This was achieved by querying our medical database each week using Current Procedural Terminology (CPT) code 20550, “injection(s) single tendon sheath, or ligament, aponeurosis (e.g., plantar fascia).”

Patients who received a corticosteroid injection for trigger finger during the data collection period from June 4, 2019, to October 17, 2019, were invited to participate in the study. Patients who declined to participate or who did not respond to the survey were excluded. The injections were performed sterilely in the clinic setting using a 25 gauge needle and a combination of 1 cc of corticosteroid and 1 cc of 1% lidocaine without epinephrine. The injections were administered directly into the palm of the hand over the A1 pulley in the palm of the hand. During the data collection period, 672 patients received injections. Three patients declined to participate in the study, and 217 patients did not respond to the survey. Therefore, a total of 452 patients made up the cohort. The average age of the cohort was 66 years (range 25-91). Two hundred sixty-one (58%) of the patients were women, 373 (83%) were right-hand dominant, and two patients were ambidextrous. There were 119 thumbs, 42 index, 144 middle, 121 ring, and 26 little fingers included.

Patients were contacted by phone at one week (W1), two weeks (W2), and three weeks (W3) after the injection to complete a questionnaire regarding their symptoms of pain and triggering. During each phone call, the caller asked a series of standardized questions. For the pain section of the questionnaire, patients were asked: (1) whether they had complete, partial, or no pain relief; (2) for those who had complete or partial pain relief, how many days post-injection did improvement begin. Similarly, for the triggering section of the questionnaire, patients were asked: (1) whether they had complete, partial, or no triggering relief; (2) for those who had complete triggering relief, how many days post-injection did improvement begin. No further calls were made once the patients reported full resolution of pain and triggering. In addition, the electronic medical records were reviewed to collect demographic data including age, sex, handedness, smoking status, medical comorbidities, laterality of injection, and finger affected, and the type of corticosteroid used.

A statistical power analysis, with power set to 80%, was performed prior to the study to determine the number of subjects needed to detect a difference (p<0.05), yielding 385 patients. Two separate logistic regressions were analyzed for this study. The first one evaluated whether or not pain was relieved, and the second one evaluated whether or not triggering was relieved. Significance was determined by any p-value <0.05. All statistical analyses were done using R Studio Version 3.6.1 (Boston, MA: R Studio).

## Results

Patient demographics are provided in Table 1. Multivariate analysis revealed that increasing age correlated with pain improvement (p=0.03). For all other variables, we found no association with pain or triggering improvement (p>0.05).

**Table 1:** Patient demographics.

Patient demographic	
Age (years)	66 (25-91)
Gender	
Male	191 (42%)
Female	261 (58%)
Handedness	
Right	378 (84%)
Left	72 (16%)
Ambidextrous	2 (1%)
Finger injected	
Thumb	119 (26%)
Index	42 (9%)
Long	144 (32%)
Ring	121 (27%)
Small	26 (6%)
Type of corticosteroid injected	
Betamethasone	240 (53%)
Celestone	212 (47%)
History of diabetes	
Yes	78 (17%)
No	374 (83%)

Pain

**Figure 1:**
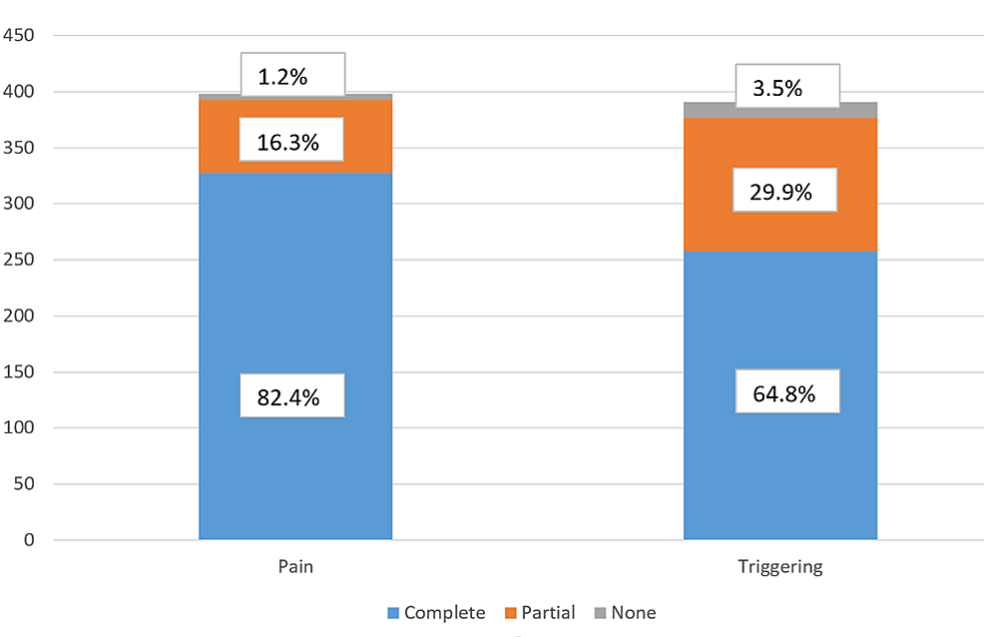
Improvement in pain and triggering at three weeks.

Of the 452 patients, 431 patients reported pain in their finger prior to injection. Overall, at W3, 328 (82.4%) patients reported complete pain relief, 65 (16.3%) had partial pain relief, and five (1.2%) had no pain relief (Figure 1).

**Figure 2:**
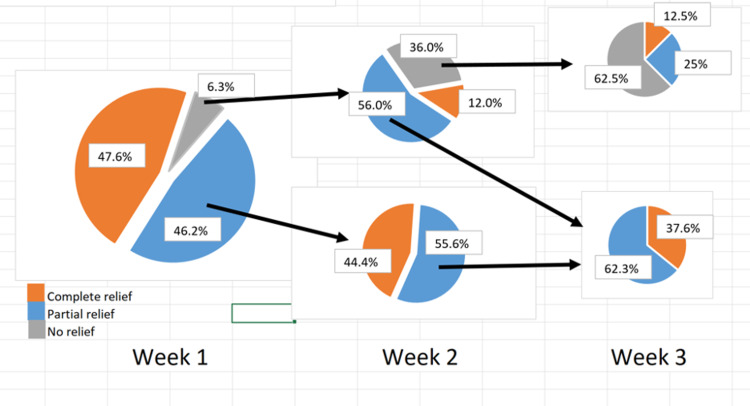
Pain relief in patients who did not experience complete relief in week 1.

Thirty-three patients did not have complete data through week 3 of the survey. At W1, 205 (47.6%) patients had complete pain relief, 199 (46.2%) had partial pain relief, and 27 (6.3%) had no pain relief. At W2, 206 of the 226 patients who still had pain after W1 reported data. Eighty-four (19.4%) had complete pain relief, 114 (26.4%) had partial pain relief, and eight (1.9%) had no pain relief. At W3, 109 of the 122 patients who still had pain after W2 reported data, with 39 (9.0%) had complete pain relief, 65 (15.0%) had partial relief, and 5 (1.2%) had no pain relief. Pain relief in patients who did not experience complete pain relief in week 1 is given in Figure 2.

In patients who experienced partial pain relief, the average time post-injection was 2.9 days (range 0-20). Patients that experienced complete pain relief did so at an average of 6.6 days (range 0-20) post-injection. For these patients, the average pain level (VAS 0-10) was 0.8 (range 0-5) at W1, and 0 at W2 and W3 post-injection. Patients that experienced partial pain relief did so at an average of 2.9 days (range 0-20) post-injection. For these patients, the average pain level was 4.6 (range 1-10) at W1, 3.4 (range 1-6) at W2, and 2.7 (range 1-7) at W3 post-injection.

From W1 to W2, 11 patients reported a worsening in pain level (on average 2.7 W1 vs. 4.8 W2). From W2 to W3, eight patients reported a worsening in pain level (on average 2.8 W1 vs. 4.1 W2). Four patients who did not report pain worsening between W1 and W2 reported worsening on W3. None of the patients who experienced partial relief regressed to no relief over the study period.

Triggering

**Figure 3:**
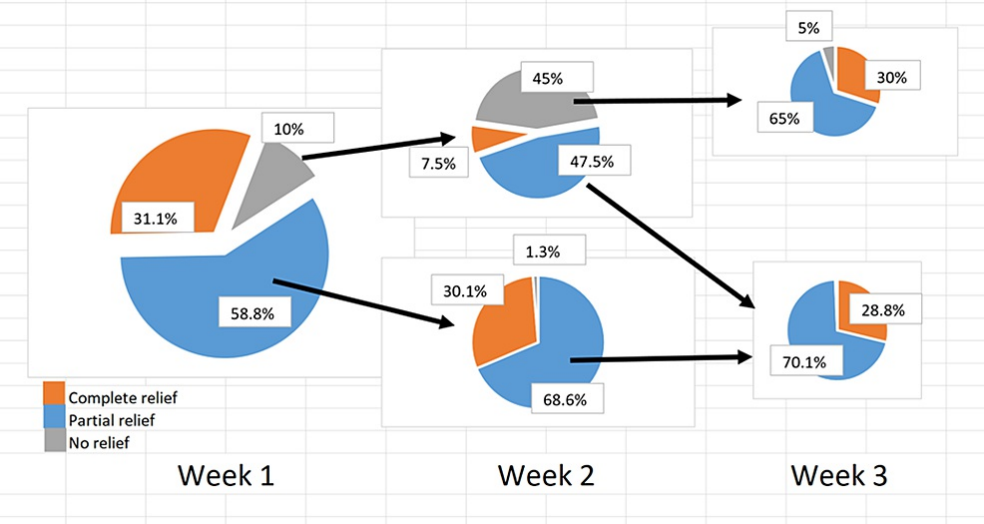
Trigger relief in patients who did not experience complete relief in week 1.

Of the 452 patients, 440 patients reported triggering in their finger prior to injection. Overall, at W3, 258 (65.9%) patients reported complete triggering relief, 119 (30.4%) had partial triggering relief, and 14 (3.5%) had no triggering relief. There were 40 patients who did not complete all surveys at the third week of follow-up. At W1, 137 (31.1%) patients had complete trigger relief, 259 (58.8%) had partial trigger relief, and 44 (10.0%) had no trigger relief. At W2, 276 of the 303 patients still had triggering after W1 reported data. 74 (16.8%) had complete trigger relief, 181 (41.1%) had partial trigger relief, and 21 (4.7%) had no trigger relief. At W3, 180 of the 202 patients still had triggering after W2 reported data, with 47 (10.6%) had complete trigger relief, 119 (27.0%) had partial relief, and 14 (3.1%) had no trigger relief. Triggering relief in patients who did not experience complete pain relief in week 1 is given in Figure 3.

Patients who experienced complete relief of pain did so at an average of 8.1 days (range 0-22) post-injection. From W1 to W2, 3 patients who initially had partial improvement reported no longer having improvement, and this regression of improvement continued into W3 for two of the patients, with the third patient reporting improvement in W3. There was one patient who reported having improvement W1 and W2 who subsequently reported no further improvement in W3.

## Discussion

Consistent with our hypothesis, we found that the majority of patients experienced some pain relief within the first week following corticosteroid injection, with approximately half achieving complete pain relief. It appears that the extent to which patients experience pain relief during this first week is predictive of the rapidity of their response to the injection. If patients do not experience any relief of pain during that first week, they are much less likely to have complete relief of pain during week 2. Furthermore, patients who do not have complete relief by the end of week 2 are unlikely to have relief by three weeks. Conversely, patients who experience a partial relief of symptoms by the end of week 1 are much more likely to have an improvement over the ensuing weeks.

In contrast to the trend seen in pain relief, the improvement in triggering seems to lag - fewer patients had an early (week 1) response and fewer patients had complete resolution of the trigger by week 3. Additionally, early improvement in triggering (or lack thereof) does not seem to be predictive of the speed of resolution. Even among the patients who did not experience complete triggering relief during the first one to two weeks, the vast majority had a partial or complete response by week 3.

While there are many studies demonstrating the long-term effectiveness of corticosteroid injections for trigger finger, there is little prospective data that documents the time course of symptom improvement [5,11,16,17-24]. In a prospective study of trigger finger corticosteroid injections, Anderson and Kaye noted typical pain relief in three to five days and resolution of mechanical locking symptoms in two to three weeks [11]. However, given that their initial follow-up evaluation was at six weeks post-injection and that no data is given to support these values, it is unclear how these times were estimated. In evaluating the incidence of flare reaction, Goldfarb et al. assessed visual analog pain scores for seven days post-injection and then again at six weeks post-injection [25]. After a brief post-injection improvement in pain from the local anesthetic, they found that the pain level increased on day 1 after injection and then slowly decreased over the next week. The timing of trigger relief was not noted in this study. In a prospective study of patients undergoing trigger finger (TF) corticosteroid injections, Julka et al. assessed pain for one day after injection. They found that pain during the injection was the only significant predictor of pain on the day after injection [26].

Given the paucity of data on this topic, our results are valuable in counseling patients prior to their corticosteroid injection. Setting appropriate expectations has the potential to increase patient satisfaction while decreasing unnecessary phone calls and/or follow-up visits in the early post-injection period. Moreover, realistic expectations are important for the injecting surgeon as well. In a study of treatment strategies for trigger fingers, Zyluk and Jagielski found that early post-injection reevaluation led to increased surgery as compared to delayed or as-needed reevaluation [27]. The overall benefits of the corticosteroid injections should be weighed against potential complications including tendon ruptures, infection, allergic reactions, etc.

There are several limitations to our study. First, the volume of injectate was not standardized or recorded across all surgeons. While the surgeons in the study tend to use a total of 1-2 mL of injectate (combination of lidocaine and corticosteroid), we did not specifically record the volume injected into each patient so there may have been some variability. However, given that the volumes injected were relatively small, we doubt that some minor variability would have had a substantial impact on the results. Second, the type of injectate was not standardized across all surgeons. Given that corticosteroids vary in their rapidity of action and duration of effect, it is possible that this may have impacted our results [28]. Third, we did not evaluate whether the injections were given into the sheath directly or were given outside of the sheath. While Taras et al. demonstrated that the overall efficacy of intra- and extra-sheath injections are comparable, it is possible that the timing of symptom improvement varies as a function of injection location with respect to the sheath [29]. Fourth, we did not contact patients beyond week 3 based on the study design. However, given that approximately 17% of patients continued to have some pain and approximately 33% of patients had persistent triggering, it may have been valuable to reassess these patients at later time points (W4, etc). Finally, we relied upon patient responses via phone to determine the nature and quality of their symptoms. There may have been some variability in the way that patients interpreted partial or complete relief, and hence some inaccuracy in what they reported.

## Conclusions

We found that most patients experience relief of pain and triggering by week 3 after corticosteroid injection, though pain improved more rapidly and reliably than triggering within this time interval. We counsel patients that improvements in symptoms after corticosteroid injection for trigger finger can be delayed and that they should not be surprised if it takes a few weeks to see an effect. Understanding the timing of the effect of the injection may help to counsel the surgeon on the timing of surgical intervention.
